# Notes and News

**Published:** 1897-06-26

**Authors:** 


### Notes and News.

(Collected and Communicated.)

In addition to her bequest to the Throat Hospital, Golden Square, the late Mrs. Ellen H. Harris has bequeathed £1,000 to the Great Ormond Street Hospital for Sick Children.

In the list of those who have received honours in connection with Her Majesty's Jubilee appears the name of Mr. Henry C. Burdett, who has been appointed Knight Commander of the Most Honourable Order of the Bath. Mr. Burdett has from the first edited The Hospital, and we offer him our most hearty congratulations.

The interest and sympathy exhibited towards the Prince of Wales's Hospital Fund has been very general. Not the least gratifying sign of this is the movement on its behalf on the part of the Italian residents in London, many of whom are working men. A committee has been formed and a collection made, resulting in a contribution of £135 to the Fund.

Preparations for the opening of the New General Hospital at Birmingham, and the Royal visit in connection with it, are fast approaching completion. Princess Christian will perform the ceremony on July 7th, directly representing the Queen on the occasion. Before the ceremony the Royal party will be entertained at luncheon by the Lord Mayor.

Following the example of the Seamen's Hospital at Greenwich, the managers of the Newcastle Royal Infirmary have taken steps to secure the exhibition of a Japanese battleship to the public on behalf of the institution. Through the kind permission of the authorities, the *Yashima* was thrown open for inspection on the 14th and 15th of the present month.

Amongst the interesting arrangements for the Jubilee was the opportunity afforded by Mr. T. H. Roberts, editor of *Illustrated Bits*, to the survivors of the Balaclava Charge to witness the Jubilee procession. Mr. Roberts' offices are in Fleet Street, and he issued an invitation to the veterans to be his guests from Monday, 21st, to the Wednesday following. Sixty-nine acceptances were received, and many letters expressing the genuine pleasure the invitation gave them, not only to see the interesting spectacle, but to meet old comrades.

Lady Rothschild has laid the foundation-stone of the new cottage hospital at Acton. The site has been given by Lord Rothschild and Mr. Leopold de Rothschild, and the cost of erecting the building will be defrayed by Mr. Passmore Edwards, after whom the institution will be named. The parishioners of Acton have contributed nearly £1,000 towards the cost of maintenance. The institution is to serve as hospital, nursing institution, and invalid kitchen.

A Medical, Surgical, and Hygienic Exhibition is to be made of all the manufactured "chemical preparations for medical purposes, surgical instruments, and appliances, food products, sanitary compounds and apparatus, and the literature bearing on the science and art of medicine and surgery." It will be held in the Queen's Hall, Langham Place, W., on July 26th—30th. The exhibition will be arranged on historical lines, showing the progress of the last sixty years, and a pathological and anatomical museum will be of special interest. Amongst the exhibits will be found one of The Scientific Press.

The following members of the medical profession are amongst those who have received honours on the occasion of the Jubilee: Sir William MacCormac, Mr. Thomas Smith, and Mr. Samuel Wilks have received the dignity of baronetcy; Dr. Edward Frankland and Mr. Richard Thorne-Thorne have been appointed Knights Commanders and Dr. T. W. Grimshaw and Mr. David Nicholson Companions of the Order of the Bath. Amongst the military K.C.B.'s are Surgeon-General A. Gordon and the C.B.'s Surgeon-Major-General C. Sibthorpe and Brigade Surgeon-Lieut.-Colonel G. McNalty; and in the Civil list of C.B.'s appears the name of Surgeon-Major-General James Jameson. In connection with the Order of the Star of India, the distinction of Companion of the Order has been conferred upon Surgeon-Major Cleghorn. Dr. William Gowers, Dr. Felix Semon, and Mr. Thomas Naghton Fitzgerald have been created knights.

The ambulance arrangements for Jubilee were very complete. Three associations shared the field—the St. John Ambulance Association, the Hospitals' Association Ambulance Service, and the Medical Staff Corps; the last-named at the service of the troops. The Hospitals' Association Ambulance Service held 24 stations, which were divided between six sections, each section being attended by a nurse. One ambulance with two bearers (commissionaires) was apportioned to each of 20 stations, and two ambulances with bearers to each of the remaining four. Every station was under the control of a medical man; and doctors, nurses, and medical men had each a card giving full particulars of the various stations and the staff on duty thereat, consequently in cases of emergency all could have acted in unison. The association were in communication with various police-stations, and thus a most efficient and complete organisation was effected in a quiet and business-like manner typical of its work on all occasions. The St. John Ambulance Association was everywhere and most active, so much so that in one instance a disabled soldier was promptly rescued from the hands of the Army Staff Corps, by whom he was being attended, and who remained in possession of his accoutrements.

